# Antioxidant and Anti-Skin Aging Potential of Selected Thai Plants: In Vitro Evaluation and In Silico Target Prediction

**DOI:** 10.3390/plants12010065

**Published:** 2022-12-22

**Authors:** Kamonwan Chaikhong, Sawarin Chumpolphant, Panthakarn Rangsinth, Chanin Sillapachaiyaporn, Siriporn Chuchawankul, Tewin Tencomnao, Anchalee Prasansuklab

**Affiliations:** 1 Graduate Program in Clinical Biochemistry and Molecular Medicine, Department of Clinical Chemistry, Faculty of Allied Health Sciences, Chulalongkorn University, Bangkok 10330, Thailand; 2 Department of Transfusion Medicine and Clinical Microbiology, Faculty of Allied Health Sciences, Chulalongkorn University, Bangkok 10330, Thailand; 3 Department of Clinical Chemistry, Faculty of Allied Health Sciences, Chulalongkorn University, Bangkok 10330, Thailand; 4 Natural Products for Neuroprotection and Anti-Ageing Research Unit, Chulalongkorn University, Bangkok 10330, Thailand; 5 College of Public Health Sciences, Chulalongkorn University, Bangkok 10330, Thailand

**Keywords:** phytomedicine, anti-aging, antioxidant, anti-elastase, anti-tyrosinase, proanthocyanidin, *Areca catechu*, *Anacardium occidentale*, *Glochidion zeylanicum*, *Senna alata*

## Abstract

The skin is the largest organ that performs a variety of the body’s essential functions. Impairment of skin structure and functions during the aging process might severely impact our health and well-being. Extensive evidence suggests that reactive oxygen species play a fundamental role in skin aging through the activation of the related degradative enzymes. Here, the 16 Thai medicinal plant species were screened for their potential anti-skin aging properties. All extracts were investigated for total phenolic and flavonoid contents, antioxidant, anti-elastase, and anti-tyrosinase activities, as well as the binding ability of compounds with target enzymes by molecular docking. Among all the plants screened, the leaves of *A. occidentale* and *G. zeylanicum* exhibited strong antioxidants and inhibition against elastase and tyrosinase. Other potential plants include *S. alata* leaf and *A. catechu* fruit, with relatively high anti-elastase and anti-tyrosinase activities, respectively. These results are also consistent with docking studies of compounds derived from these plants. The inhibitory actions were found to be more highly positively correlated with phenolics than flavonoids. Taken together, our findings reveal some Thai plants, along with candidate compounds as natural sources of antioxidants and potent inhibitors of elastase and tyrosinase, could be developed as promising and effective agents for skin aging therapy.

## 1. Introduction

Skin is the largest and most complex organ in the human body. The skin serves as a barrier between the body and the outside environment, and it serves a variety of functions [1]. It has a significant cosmetic role in addition to protecting the body from water loss and microbial infection [2]. In addition, it works to support other body parts, such as the immune, nervous and endocrine systems [1]. The look of youth and beauty may have a positive impact on people’s social behavior and human life [1,2]. Hence, the impairment of skin structure and functions that occur as we age might have a severe impact on our health and well-being [2,3]. Thinness, dryness, lack of elasticity, rough texture, wrinkles, and dark pigments are all common characteristics of older skin [4]. Many researchers are currently working on generating potential anti-aging drugs or chemicals, particularly those derived from natural sources for skin aging treatment.

In general, human skin ages in two ways: internally (as a result of chronological aging) and extrinsically (as a result of environmental variables influenced by environmental factors) [5]. Extensive evidence suggests that oxidative stress, through the formation of reactive oxygen species (ROS), plays a fundamental role in both intrinsic and extrinsic skin aging [6]. ROS causes oxidative damage to skin cells by damaging essential macromolecules such as nucleic acids, enzymatic proteins, and membrane lipids, resulting in cellular malfunction and cell death [7]. Oxidative stress also contributes to the degradation of the extracellular matrix (ECM) by suppressing ECM component synthesis (e.g., elastin) and activating ECM degrading enzymes (e.g., elastase), which results in loss of skin elasticity [2,8,9]. Moreover, ROS can cause irregular or dark colors in the skin by inducing the production of an α-melanocyte-stimulating hormone (α-MSH) in keratinocytes, thereby triggering the activation of the tyrosinase enzyme and promoting melanin synthesis in melanocytes [10,11]. Therefore, scavenging ROS and inhibiting elastase and tyrosinase activities could be useful in the treatment or even prevention of skin aging.

Natural products are currently receiving much interest as potential alternative medicines for treating a number of diseases as well as aging and age-associated declines [8,12,13]. Tropical plants could be of interest to explore their potential in skin aging treatments. As reported previously, several Andean and Himalayan plants have been regarded as sources of compounds with potential use as anti-aging ingredients [14,15]. Thailand is a known place for cultivating a wide variety of tropical plants, many of which have not been studied extensively. The goal of this research was to find potential natural sources for developing novel treatments against skin aging. The extracts of 16 Thai medicinal plant species were studied in vitro for their properties related to anti-skin aging, including total phenolic and flavonoid contents, free radical scavenging, anti-elastase, and anti-tyrosinase activities. We also further performed correlation analysis and an in silico molecular docking approach to reveal the promising phytochemical compounds in the three most effective plants with strong inhibition against elastase or tyrosinase enzyme.

## 2. Results

### 2.1. Extraction Yields

Table 1 shows the scientific name, parts used, source, extraction method/solvent, and percent yield of Thai plants used in this study. The percent yields of the extracts ranged from 2.0% to 36.3% (Table 1). *C. carandas* had the highest extraction yield (36.3%), followed by *M. caloneura* (18.2%) and *A. occidentale* (16.0%), whereas *H. undatus* had the lowest yield (2.0%).

### 2.2. Total Phenolic Content of Thai Plants

The plant extracts showed a variety of total phenolic content ranging from 14.06 ± 1.55 to 320.14 ± 7.95 mg of gallic acid equivalent (GAE) per g dry weight extract (Table 2). *G. zeylanicum* (320.14 ± 7.95 mg GAE per g dry weight extract) had the highest phenolic content in all extracts, followed by *A. catechu* (295.79 ± 11.97 mg GAE per g dry weight extract) and *M. caloneura* (210.99 ± 10.40 mg of GAE per g dry weight extract), respectively. The lowest level was found in *C. carandas* at 14.06 ± 1.55 mg GAE per g dry weight extract.

### 2.3. Total Flavonoid Content of Thai Plants

The total flavonoid content of plant extracts varied among the plant species, ranging from 2.61 ± 0.42 to 84.48 ± 18.32 mg of quercetin equivalent (QE) per g dry weight extract (Table 2). Of all extracts, the highest flavonoid content was found in *M. caloneura* (84.48 ± 18.32 mg of QE per g dry weight extract), followed by *G. zeylanicum* (52.54 ± 7.25 mg of QE per g dry weight extract) and *P. dulce* (28.01 ± 4.39 mg of QE per g dry weight extract), respectively. On the other hand, the lowest level was found in *E. americana* at 2.61 ± 0.42 mg of QE per g dry weight extract.

### 2.4. DPPH Radical Scavenging Activity of Thai Plants

The DPPH assay is based on the hydrogen-donating capacity of the compound to scavenge the stable DPPH radicals [16]. The antioxidant capacities of plant extracts were expressed as the percent DPPH radical scavenging activity, the mg of vitamin C equivalent antioxidant capacity (VCEAC) per g dry weight extract, and the half-maximal inhibitory concentration (IC_50_) (Table 3). At 0.1 mg/mL of extracts, the percentages of DPPH scavenging activity ranged from 6.40 to 93.18%. Four plant extracts: *G. zeylanicum* (93.18%), *M. caloneura* (90.61%), *A. occidentale* (89.01%), and *A. catechu* (88.12%), exhibited DPPH scavenging activity greater than 80%. In contrast, *C. ternatea* showed the lowest scavenging activity at 6.40%. This rank order was the same when compared based on the relative VCEAC values. However, according to the IC_50_ values, the antioxidant capacity of the top four plant extracts was changed in the following order: *G. zeylanicum* > *A. catechu* > *A. occidentale* > *M. caloneura*. The obtained results of the DPPH assay show that *G. zeylanicum* exhibited the strongest antioxidant potential with the highest percentage of scavenging activity and the lowest IC_50_ value.

### 2.5. ABTS Radical Scavenging Activity of Thai Plants

The ABTS assay is based on the compound’s ability to transfer hydrogen atoms for neutralizing a stable ABTS radical cation. The antioxidant capacities of plant extracts were expressed as the percent ABTS radical scavenging activity, the mg of VCEAC per g dry weight extract, and the IC_50_ (Table 4). At 0.1 mg/mL of extracts, the percentages of ABTS scavenging activity ranged from 13.93% to 99.37%. Eight plant extracts: *G. zeylanicum* (99.37%), *A. catechu* (99.31%), *A. occidentale* (99.23%), *M. caloneura* (98.88%), *E. americana* (95.62%), *Z. officinale* (94.07%), *P. dulce* (83.99%), and *H. undatus* (82.16%) exhibited the ABTS scavenging activity greater than 80%. In contrast, *C. carandas* showed the lowest scavenging activity at 13.93%. The antioxidant capacity of the top four plant extracts, according to the VCEAC, and IC_50_ values were found in a similar rank order as follows: *G. zeylanicum* > *A. catechu* > *A. occidentale* > *M. caloneura*. Remarkably, the results of the ABTS assay also showed that the extract with the strongest antioxidant activity was *G. zeylanicum* leaf, as represented by the highest percentage of scavenging activity and the lowest IC_50_ value.

### 2.6. Anti-Elastase Activity of Thai Plants

The elastase inhibitory activity of plant extracts was evaluated using the elastase inhibition assay with N-succinyl-trialanyl-paranitroanilide (SANA) as the substrate. Epigallocatechin gallate (EGCG) (0.1 mg/mL), which was used as a positive control, showed an inhibition level of 45.27%. The elastase inhibitory activities of the extracts are presented in Table 5 (see also Table S1). At 0.5 mg/mL of extracts, the percentages of elastase inhibition ranged from 1.33% to 88.31%. *A. catechu* had the highest elastase inhibitory effect at 88.31%, followed by *G. zeylanicum* (87.43%), *A. occidentale* (84.78%), and *S. alata* (73.95%), while *S. asper* bark had the lowest elastase inhibitory effect at 1.33%. Due to high background absorbance, some plant extracts with no detectable activity were reassayed at 0.1 mg/mL. It was found that *M. caloneura*, *P. nigrum*, *Z. cassumunar,* and *S. asper* leaf, except *Z. officinale,* showed low to moderate inhibitory activities ranging from 3.46% to 35.74%. Nevertheless, the effects of *C. carandas*, *C. asiatica*, *C. ternatea*, *E. americana*, and *H. undatus* were not observed even at higher concentrations.

### 2.7. Anti-Tyrosinase Activity of Thai Plants

Tyrosinase inhibitory activity of plant extracts was evaluated using the dopachrome method with 3,4-dihydroxy-L-phenylalanine (L-DOPA) as the substrate. Kojic acid (KA) (0.02 mg/mL), a positive control, showed an inhibition level of 68.35%. The tyrosinase inhibitory activity of the extracts is presented in Table 6 (see also Table S2). At 1 mg/mL of extracts, the percentages of tyrosinase inhibition ranged from 4.80% to 91.51%. *G. zeylanicum* had the highest tyrosinase inhibitory effect at 91.51%, followed by *A. occidentale* (81.01%), *M. caloneura* (76.12%), and *A. catechu* (75.38%), whereas *P. nigrum* had the lowest tyrosinase inhibitory effect at 4.80%. Two plant extracts with no detectable activity due to high background absorbance were reassayed at 0.1 mg/mL, and the activities were found at 3.98% (*Z. cassumunar*) and 21.28% (*Z. officinale*). However, *P. dulce* did not exhibit any effect, even at increased concentration.

### 2.8. Correlation Analysis

**Figure 1:**
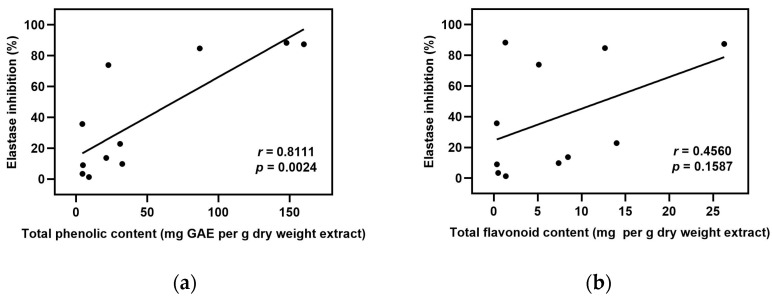

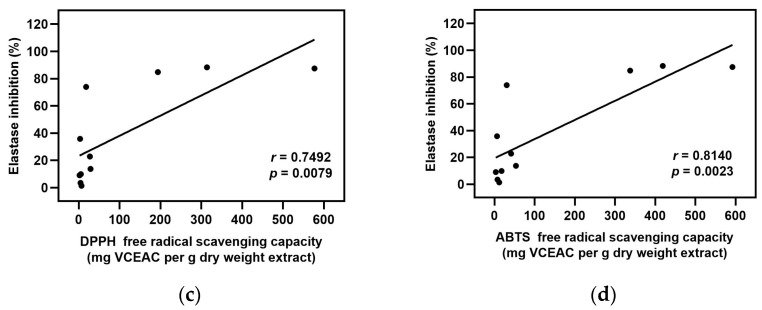
Correlation analysis between elastase inhibition and values in Table 1, Table 2, Table 3 and Table 4 were expressed as Pearson’s correlation coefficients (*r*): (**a**) elastase inhibition versus total phenolic content; (**b**) elastase inhibition versus total flavonoid content; (**c**) elastase inhibition versus DPPH free radical scavenging activity; (**d**) elastase inhibition versus ABTS free radical scavenging activity.

**Figure 2:**
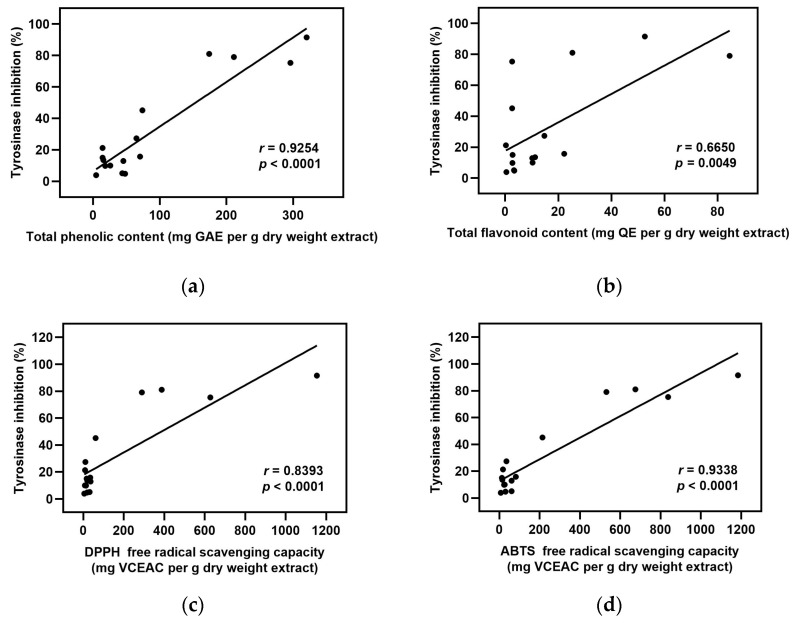
Correlation analysis between tyrosinase inhibition and values in Table 1, Table 2, Table 3 and Table 4 were expressed as Pearson’s correlation coefficients (*r*): (**a**) tyrosinase inhibition versus total phenolic content; (**b**) tyrosinase inhibition versus total flavonoid content; (**c**) tyrosinase inhibition versus DPPH free radical scavenging activity; (**d**) tyrosinase inhibition versus ABTS free radical scavenging activity.

Considering several previous reports of potential elastase and tyrosinase inhibitory action of plant extracts [8,16,17], it was likely that the extracts possess a high antioxidant potential and tend to have strong elastase and tyrosinase inhibitory action. Thus to verify those relationships in this study, we further performed Pearson’s correlation analysis to investigate the relationship between the inhibitory activities of both enzymes and the level of antioxidant contents as well as antioxidant capacities among the extracts used. The strength of the correlation is distributed by correlation coefficient (*r*-value) as follows: *r* = 0.910 to 1.000 indicate a very strong correlation, *r* = 0.710 to 0.900 indicate a high correlation, *r* = 0.410 to 0.700 indicate a moderate correlation, *r* = 0.210 to 0.400 indicate a small correlation, and *r* = 0.000 to 0.200 indicate a slight correlation [18]. The elastase inhibition had a high positive correlation to total phenolic content, DPPH, and ABTS radical scavenging activities, as shown in Figure 1a,c,d, respectively. Similarly, the tyrosinase inhibition showed a very strong positive correlation to total phenolic content and ABTS radical scavenging activity (Figure 2a,d), while it showed a high positive correlation to DPPH radical scavenging activity (Figure 2c). However, the total flavonoid content was moderately positively correlated to both elastase and tyrosinase inhibition (Figure 1b and Figure 2b). These results demonstrated that phenolic compounds and antioxidant activity might have a significant contribution to the inhibition of elastase and tyrosinase enzymes.

### 2.9. Molecular Docking

We next evaluated the abilities of phytochemical compounds in the three most effective plants with strong inhibition against elastase or tyrosinase enzymes. Molecular docking is generally used to predict the binding affinity of compounds to protein receptors or enzymes compared to known inhibitors. According to our results of in vitro screening assays, we found that *A. occidentale* was the most potent elastase inhibitor, followed by *G. zeylanicum* and *S. alata*, in rank order of IC_50_ values. Thus, we selected phytochemical compounds derived from these three plants to evaluate their ability to inhibit elastase. The results of interactions between elastase (3HGP) and compounds are presented in Table S4 as binding energy, inhibition constant, the number of hydrogen bonds, amino acid interaction, and bond length. The binding energy between 3HGP and compounds shows scores ranging from −2.26 to −11.95 kcal/mol (Table S4). EGCG, a positive control, showed the binding energy at −9.69 kcal/mol. According to the docking results, five compounds showed lower binding energy than the positive control. Tetramer of proanthocyanidin exhibited the lowest binding energy (−11.95 kcal/mol), which indicated that it has the best affinity compared to other compounds in elastase inhibition, followed by amentoflavone, rutin, agathisflavone, and kaempferol 3-O-gentiobioside, with the binding energies at −11.81, −10.12, −9.92, and −9.73, respectively.

**Figure 3:**
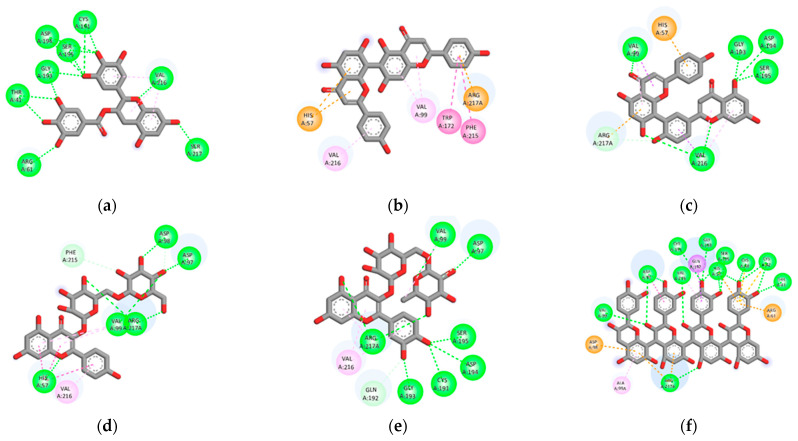
Molecular docking analysis of elastase (3HGP) is represented by the 2D diagrams of interaction between 3HGP and positive control and compounds with the lowest binding energy: (**a**) epigallocatechin gallate (positive control); (**b**) agathisflavone; (**c**) amentoflavone; (**d**) kaempferol 3-O-gentiobioside; (**e**) rutin; (**f**) tetramer of proanthocyanidin.

**Figure 4:**
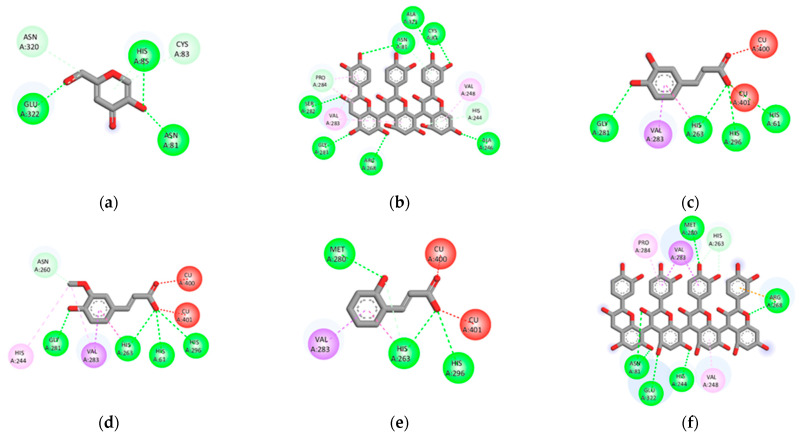
Molecular docking analysis of tyrosinase (2Y9X) is represented by the 2D diagrams of interaction between 2Y9X and positive control and compounds with the lowest binding energy: (**a**) KA (positive control); (**b**) anacardic acid triene; (**c**) caffeic acid; (**d**) ferulic acid; (**e**) o-coumaric acid; (**f**) tetramer of proanthocyanidin.

In addition, the rank order of IC_50_ values for plant extracts with tyrosinase inhibition was closely similar to elastase inhibition. We found that *G. zeylanicum* possessed the highest inhibitory effect, followed by *A. catechu,* and *A. occidentale*. Hence, the compounds derived from these three plants were selected to investigate their ability to inhibit tyrosinase. The results of interactions between tyrosinase (2Y9X) and compounds are presented in Table S5 as binding energy, inhibition constant, the number of hydrogen bonds, amino acid interaction, and bond length. The binding energy between 2Y9X and compounds showed scores ranging from −4.56 to −10.42 kcal/mol (Table S5). KA, a well-known inhibitor of tyrosinase, showed the binding energy at −4.59 kcal/mol. Based on the docking results, o-coumaric acid (−10.42 kcal/mol) and tetramer of proanthocyanidin (−10.42 kcal/mol) exhibited the lowest binding energy against elastase when compared to other compounds, followed by caffeic acid, ferulic acid, and arecatannin A1 with the binding energies at −10.10, −10.00, and −9.94, respectively. Figure 3 and Figure 4 represent the 2D diagrams of ligand–protein interactions for elastase and tyrosinase, for the positive control, and for five compounds with the lowest binding energy.

## 3. Discussion

Skin aging is a naturally occurring process in all human beings. However, many lifestyles and environmental factors can also accelerate this process leading to prematurely aged skin [19]. ROS is well known as an important pathogenic factor in the aging process of the skin. The accumulation of ROS can upregulate the expression of both elastase and tyrosinase enzymes, which subsequently leads to wrinkle formation, lack of elasticity, and hyperpigmentation [20,21,22]. All of these are common characteristics of skin aging [4]. Here, our study investigated the antioxidant, anti-elastase, and anti-tyrosinase properties of plant extracts from 16 Thai plant species and revealed promising natural compounds for the potential development of novel treatments against skin aging.

Elastase is a protease enzyme that is primarily responsible for the degradation of elastin, an important protein found in the ECM. Elastin is vital for giving elasticity to the skin due to its elastic recoil properties [13]. Therefore, the inhibition of elastase activity can be helpful in preventing skin loss of elasticity and wrinkles [23]. Our result found that *A. occidentale* was the most potent elastase inhibitor, followed by *G. zeylanicum* and *S. alata*, in rank order of IC_50_ values. Furthermore, the docking results revealed that five compounds derived from these three plants have lower binding energies than EGCG (positive control) and FRW (original inhibitor), wherein the compounds displaying the lower binding energy were considered to have better inhibition (Table S4) [24]. Those compounds are flavonoids, which include a tetramer of proanthocyanidin [25], amentoflavone [25], rutin [26,27], agathisflavone [26] from *A. occidentale*, and kaempferol 3-O-gentiobioside from *S. alata* [28] (Table S3). The results suggested all five compounds to be responsible for anti-elastase activity as well as can be regarded as promising candidates for the development of anti-skin aging. However, we found that none of the compounds from *G. zeylanicum* showed strong binding affinity as compared to the control ligands, although its extract showed the second most activity by in vitro assay. This may be due to the synergistic effect of compounds in the mixture rather than the individual effect of each phytochemical component.

In addition to elastase, melanin is considered another important target for skin-aging treatment. Melanin is a major component of the skin, hair, and eye color synthesized by melanogenesis within the melanocyte. However, overproduction of melanin may cause skin disorders, including freckles, melasma, age spots, and hyperpigmentation, leading to a premature aging appearance [12]. In melanogenesis, tyrosinase is the critical enzyme in the rate-limiting step. Therefore, the downregulation of tyrosinase activity can lead to reduced melanin production [29]. Our results showed that *G. zeylanicum* was the most potent tyrosinase inhibitor, followed by *A. catechu* and *A. occidentale*, according to IC_50_ values. Surprisingly, most of the compounds (47 of 48 compounds) in these three plants showed lower binding energy against tyrosinase than KA (positive control) and tropolone (original inhibitor) (Table S5). Among the 47 compounds, 16 were derived from *G. zeylanicum*, 12 were derived from *A. catechu*, and 29 were derived from *A. occidentale*, of which 10 of them can be found in more than one plant (Table S3). However, in contrast to the binding of the compound with elastase, the identified potential compounds against tyrosinase are from a variety of phytochemical classes. Regarding the rank of binding energies towards tyrosinase, the compounds that are considered the best five inhibitors are o-Coumaric acid [30] (phenolic), tetramer of proanthocyanidin [25] (flavonoid), caffeic acid [30] (phenolic), ferulic acid [30] (phenolic), and arecatannin A1 [31] (tannin), in the increasing order of scores (Table S5). The results obtained from in vitro screening and docking analysis in this study have confirmed the anti-skin-aging properties of four Thai medicinal plants and suggested their potential derived compounds that could be responsible for the observed activities. Further studies on fractionation, as well as the identification and isolation of bioactive compounds in these promising plant extracts, are critically required to prove the presence of our proposed molecules that could subsequently be developed for the treatment of aging skin [14].

*A. catechu*, *A. occidentale*, *G. zeylanicum*, and *S. alata* are plants used in traditional medicine and found in the tropical zone of Southeast Asia, including Thailand [32,33,34,35,36]. Notably, these plants were demonstrated for several antioxidant-related activities. *A. catechu* fruit is a popular chewable item with betel leaves, which is intoxicating and slightly addictive [36]. It is used for the treatment of burn wounds and skin ulcers and acts as an astringent [35,37]. It has been reported for potent antioxidant and anti-inflammatory effects against oxidative stress-induced liver injury in rats [38]. *A. occidentale* and *G. zeylanicum,* belonging to Southern Thailand, are used as food and local medicinal plant [32,33]. *A. occidentale* leaves are used to treat skin rashes, itching, ulcers, and fever, whereas *G. zeylanicum* leaves are used to treat rheumatoid arthritis, influenza, dysentery, and dyspepsia [32,33,39]. The leaf extracts of *A. occidentale* and *G. zeylanicum* exhibited neuroprotective effects against glutamate and H_2_O_2_-induced oxidative damage [32,40]. Moreover, crude extract from the leaves of both plants exerted antioxidative stress and anti-aging properties in the nematode *Caenorhabditis elegans* [33,41,42]. Leaves of *S. alata* are used for the treatment of skin rashes, mycosis, and dermatitis [34]. Leaf extract of this plant was able to increase both enzymatic and nonenzymatic antioxidant systems and prevent the liver and renal tissues from damage caused by oxidative stress during diabetes in a rat model [43].

In agreement with previous reports, our findings reveal that *A. catechu*, *A. occidentale*, *G. zeylanicum*, and *S. alata* exhibit antioxidant potential, apart from the activities toward skin aging-related enzymes. We found that these plants are rich in total phenolics and flavonoids with high antioxidant capacities towards DPPH and ABTS radicals, except for *S. alata*, which showed only a moderate level in both amounts and activities. Phenolics and flavonoids are two well-known classes of plant secondary metabolites that are majorly responsible for antioxidant activity [44,45]. This was consistent with our results that total phenolic and flavonoid contents demonstrated a significant positive correlation with free radical scavenging activities (Figure S1). Interestingly, the correlation analysis also revealed that the contents of total phenolic and flavonoid compounds in this studied plant extracts were positively correlated with both elastase and tyrosinase inhibition. However, the correlation strength was found to be higher with phenolics than with flavonoids. Free radical scavenging activities were shown to have a high correlation to the enzyme-inhibitory activities of the extracts. These results suggested that high phenolic content and antioxidant activity may lead to strong inhibition of elastase and tyrosinase enzymes. However, the possibility of protein-polyphenol interactions should also be a concern. Some polyphenols could directly cause enzyme precipitation via their ability to bind with proline-rich proteins, resulting in hydrogen-bond formation with the enzyme and thereby leading to non-selective inhibition [46].

## 4. Materials and Methods

### 4.1. Chemicals and Reagents

Folin–Ciocalteu’s phenol reagent, aluminum chloride (AlCl_3_), dimethyl sulfoxide (DMSO), sodium acetate (NaOAc), quercetin, 2,2-diphenyl-1-picrylhydrazyl (DPPH), 2,2′-azinobis-(3-ethylbenzthiazoline-6-sulphonic acid) (ABTS), L-ascorbic acid, potassium persulfate (K_2_S_2_O_8_), elastase from porcine pancreas, epigallocatechin gallate (EGCG), N-succinyl-Ala-Ala-Ala-p-nitroanilide (SANA), tyrosinase from mushroom, kojic acid (KA), and 3,4-dihydroxy-L-phenylalanine (L-DOPA) were purchased from Sigma-Aldrich (St. Louis, MO, USA). Sodium carbonate (Na_2_CO_3_) was purchased from Merck (Darmstadt, Germany). Gallic acid was purchased from TCI America (Portland, OR, USA). Dipotassium phosphate (K_2_HPO_4_) and monobasic potassium phosphate (KH_2_PO_4_) were purchased from HiMedia (Mumbai, India). Tris base was purchased from Vivantis Technologies (Shah Alam, Malaysia). Ethanol and methanol were purchased from RCI Labscan (Bangkok, Thailand). All chemicals and reagents were analytical grades.

### 4.2. Plant Materials and Extraction

The plants in this study were collected locally from gardens or purchased from local markets as appropriate. Table 1 provides the scientific name, part used, and source of each plant. These plants were botanically authenticated, and their voucher specimens were deposited in the herbarium of Kasin Suvatabhandhu, Department of Botany, Faculty of Science, Chulalongkorn University, Bangkok, Thailand, or identified by a botanist. The plant materials were washed, dried at 65 °C, and ground finely in a mechanical grinder. The extraction of the dried plant (40 g) was carried out by Soxhlet extraction or maceration method using 400 mL of ethanol or methanol. The extracts were filtered and evaporated to dryness under a vacuum. Then, the dried residues were dissolved in DMSO as a 100 mg/mL stock solution and stored at −20 °C for further study.

### 4.3. Determination of Total Phenolic Content

The total phenolic content was performed using the Folin–Ciocalteu method [47]. Briefly, 50 µL of extracts at 1 mg/mL in deionized water was mixed with 50 µL of 10% (*w*/*v*) Folin–Ciocalteu’s phenol reagent in a 96-well plate and incubated in the dark at room temperature (RT) for 20 min. After the incubation, 50 µL of 7.5% (*w*/*v*) Na_2_CO_3_ was added to the mixture and incubated for a further 20 min. The absorbance was measured with a microplate reader at 760 nm. The total phenolic content was calculated from a standard calibration curve using gallic acid from 1.56 to 100 µg/mL, and the results are shown as mg of gallic acid equivalent (GAE) per g dry weight extract.

### 4.4. Determination of Total Flavonoid Content

The total flavonoid content was performed using aluminum chloride (AlCl_3_) [47]. Briefly, 50 µL of extracts at 1 mg/mL in deionized water was made up to 200 µL with 95% ethanol, and then 10 µL of 10% AlCl_3_ and 10 µL of 1 M NaOAc were added to a 96-well plate. The plate was incubated in the dark at RT for 40 min, and absorbance was measured with a microplate reader at 415 nm. The total flavonoid content was calculated from a standard calibration curve using quercetin from 1.56 to 100 µg/mL, and the results showed as mg of quercetin equivalent (QE) per g dry weight extract.

### 4.5. Determination of DPPH Radical Scavenging Activity

DPPH radical scavenging activity assay was performed as described previously [47]. The DPPH^•^ working reagent was prepared by DPPH dissolved in absolute ethanol. Briefly, 180 µL of DPPH^•^ working solution was mixed with 20 µL of extracts in a 96-well plate and was incubated in the dark at RT for 15 min, and absorbance was measured with a microplate reader at 517 nm. Ascorbic acid from 1.56 to 100 μg/mL served as a standard. The radical scavenging activity was calculated as the percent inhibition of free radicals using the following equation:

(1) $$\%\;Inhibition=\frac{\left(Abs\;of\;control-Abs\;of\;sample\right)}{Abs\;of\;control}\times 100$$

Percentages of DPPH scavenging activity of each plant extract were compared with those of ascorbic acid. The results were expressed as mg of vitamin C equivalent antioxidant capacity (VCEAC) per g dry weight extract. The IC_50_ (half-maximal inhibitory concentration) was determined from the graph of percent inhibition against the concentration of each extract.

### 4.6. Determination of ABTS Radical Scavenging Activity

ABTS radical scavenging activity assay was performed as described previously [47]. The ABTS^•+^ working reagent was prepared by mixing 7 mM ABTS^•^ and 2.45 mM K_2_S_2_O_8_ at a ratio of 1:1, and the mixture had to remain for 16–18 h in the dark at RT. The ABTS^•+^ working solution was diluted with absolute ethanol for the absorbance to reach between 0.7 and 0.8 at 734 nm. Briefly, 180 µL of ABTS^•+^ working solution was mixed with 20 µL of extracts in a 96-well plate and was incubated in the dark at RT for 30 min, and absorbance was measured with a microplate reader at 734 nm. Ascorbic acid from 1.56 to 100 μg/mL served as a standard. The radical scavenging activity was calculated as the percent inhibition of free radicals using the Equation (1).

Percentages of ABTS scavenging activity of each plant extract were compared with those of ascorbic acid. The results expressed as mg of vitamin C equivalent antioxidant capacity (VCEAC) per g dry weight extract. The IC_50_ was determined from the graph of percent inhibition against the concentration of each extract.

### 4.7. Determination of Anti-Elastase Activity

The anti-elastase activity was evaluated by the elastase inhibition assay using the modified protocol [13,48,49]. Briefly, 20 µL of extracts, 10 µL of 0.4 U/mL pancreatic porcine elastase (PPE), and 140 µL of 0.1 M Tris-HCL buffer at pH 8.0 were added in 96-well plate and pre-incubated at RT for 20 min. After incubation, 30 µL of 2 mM SANA was added to the reaction mixture and further incubated for 30 min at RT. The absorbance was measured with a microplate reader at 734 nm. EGCG was used to serve as a positive control for inhibition. The negative control contained 100% DMSO instead of the extracts. The percent inhibition of elastase activity was calculated using the equation (1). The IC_50_ was determined from the graph of percent elastase inhibition against a concentration of each extract.

### 4.8. Determination of Anti-Tyrosinase Activity

The anti-tyrosinase activity was performed using the dopachrome method with some modifications [8]. Briefly, 20 µL of extracts, 20 µL of 200 U/mL mushroom tyrosinase, and 140 µL of 0.1 M phosphate buffer at pH 6.8 were added in 96-well plates and pre-incubated in RT for 20 min. After incubation, 40 µL of 2.5 mM L-DOPA was added to the reaction mixture and further incubated for 20 min at RT. The absorbance was read with a microplate reader at 492 nm. KA was used to serve as a positive control for inhibition. The negative control contained 100% DMSO instead of the extracts. The percent inhibition of tyrosinase activity was calculated using the equation (1). The IC_50_ was determined from the graph of percent tyrosinase inhibition against a concentration of each extract.

### 4.9. Molecular Docking

#### 4.9.1. Ligand Preparation

A list of phytochemical compounds from the three most effective plants with strong inhibition against elastase or tyrosinase was selected from the published literature [25,26,27,28,30,31,32,42,50,51,52,53,54,55,56,57,58,59,60,61] (Table S3). All chemical structures of the compounds were generated from the IUPAC name using BIOVIA Draw 2019 (BIOVIA, San Diego, CA, USA). Then, the compounds were cleaned geometry and saved the file to format pdb using Discovery Studio Visualizer (BIOVIA, San Diego, CA, USA). These files were converted to format pdbqt using AutoDockTools-1.5.6 software (The Scripps Research Institute, San Diego, CA, USA). 

#### 4.9.2. Protein Preparation

The X-ray crystallographic structures of elastase (PDB ID: 3HGP) [62] and tyrosinase (PDB ID: 2Y9X) [63] were obtained from RCSB Protein Data Bank. Before the docking study, using Discovery Studio Visualizer, water molecules and the original inhibitor were removed from the protein structure, excluding Cu^2+^ in structures of tyrosinase. These protein structures were prepared using the prepared protein setup in AutoDockTools-1.5.6 software. All missing hydrogens and Kollman charges were added to the protein structure and saved in the file to format pdbqt for docking study.

#### 4.9.3. Molecular Docking

Molecular docking studies were performed using the default protocol in AutoDockTools-1.5.6 software. Grid sites were set with a spacing of 0.375 Å. The x–y–z dimensions were set to 40 × 40 × 40 points for elastase and 60 × 60 × 60 points for tyrosinase. Grid box of the x, y, and z centers were 12.58, 9.36, and 2.251 for elastase and −10.044, −28.706, and −43.443 for tyrosinase. The docking study was performed using the Lamarckian Genetic Algorithm (GA) with default parameters, and docking results were analyzed using AutoDockTools-1.5.6 software and Discovery Studio Visualizer.

### 4.10. Statistical Analysis

All experiments were performed in at least triplicate, and the results were represented as the mean ± standard deviation (SD). The IC_50_ values were analyzed using SigmaPlot version 12.0 software. Correlation between different variables was expressed as Pearson’s correlation coefficients (r). The correlation was determined by using GraphPad Prism (GraphPad Software Inc., San Diego, CA, USA), and the results were considered statistically significant when *p* was less than 0.05 (*p* < 0.05).

## 5. Conclusions

In summary, our findings revealed four Thai medicinal plants that contain promising candidate compounds for development as anti-skin aging agents. The leaf extracts of *A. occidentale* and *G. zeylanicum* demonstrated strong inhibitory action against both elastase and tyrosinase enzymes, whereas the extracts of *S. alata* leaf and *A. catechu* fruit exhibited their activity more strongly only towards elastase or tyrosinase, respectively. Several compounds derived from these plants were also confirmed for their abilities to bind to both enzymes through molecular docking study. Moreover, *G. zeylanicum* leaf, *A. occidentale* leaf, and *A. catechu* fruit possess significant antioxidant potential towards free radicals with high amounts of phenolics and flavonoids. Taken together, *A. catechu* fruit, *A. occidentale* leaf, *G. zeylanicum* leaf, and *S. alata* leaf are identified as natural sources of antioxidants, anti-elastase, and anti-tyrosinase, which are considered potentially useful for the treatment against aging of the skin.

## Figures and Tables

**Table 1:** Scientific name, parts used, source, and percent yield of Thai plants.

Scientific Name	Part Used	Source	Voucher Number	Extraction Method/Solvent	%Yield (*w*/*w*)
*Anacardium occidentale* L.	Leaf	Songkhla, Thailand	015863 (BCU)	Soxhlet/Methanol	16.0
*Areca catechu* L.	Fruit	Surat Thani, Thailand	016434 (BCU)	Soxhlet/Ethanol	10.5
*Carissa carandas* L.	Fruit	Chachoengsao, Thailand	016531 (BCU)	Soxhlet/Ethanol	36.3
*Centella asiatica* (L.) Urb.	Leaf	Bangkok, Thailand	016426 (BCU)	Maceration/Ethanol	4.1
*Clitoria macrophylla* Wall.	Flower	Bangkok, Thailand	- ^a^	Soxhlet/Ethanol	11.9
*Clitoria ternatea* L.	Flower	Chonburi, Thailand	- ^a^	Soxhlet/Methanol	12.8
*Eleutherine americana* (Aubl.) Merr.	Rhizome	Ubon Ratchathani, Thailand	016530 (BCU)	Maceration/Ethanol	3.3
*Glochidion zeylanicum* (Gaertn.) A. Juss.	Leaf	Songkhla, Thailand	016061 (BCU)	Soxhlet/Methanol	11.8
*Hylocereus undatus* (Haw.) Britt. Rose.	Peel	Nonthaburi, Thailand	016446 (BCU)	Soxhlet/Ethanol	2.0
*Mangifera caloneura* Kurz.	Leaf	Songkhla, Thailand	016445 (BCU)	Soxhlet/Ethanol	18.2
*Piper nigrum* L.	Seed	Bangkok, Thailand	016428 (BCU)	Maceration/Ethanol	5.0
*Pithecellobium dulce* (Roxb.) Benth.	Peel	Chachoengsao, Thailand	017139 (BCU)	Soxhlet/Ethanol	6.4
*Senna alata* (L.) Roxb.	Leaf	Ubon Ratchathani, Thailand	016298 (BCU)	Maceration/Ethanol	10.5
*Streblus asper* Lour.	Bark	Rayong, Thailand	013419(BCU)	Maceration/Ethanol	2.5
*Streblus asper* Lour.	Leaf	Rayong, Thailand	013419(BCU)	Maceration/Ethanol	4.0
*Zingiber cassumunar* Roxb.	Rhizome	Rayong, Thailand	013701 (BCU)	Maceration/Ethanol	6.4
*Zingiber officinale* Roscoe.	Rhizome	Bangkok, Thailand	016425 (BCU)	Maceration/Ethanol	5.2

^a^ = identified by the botanists.

**Table 2:** Total phenolic and flavonoid contents of Thai plants.

Scientific Name	Part Used	Total Phenolic Content (mg GAE/g Dry Weight Extract)	Total Flavonoid Content (mg QE/g Dry Weight Extract)
*Anacardium occidentale* L.	Leaf	173.86 ± 4.75	25.34 ± 2.88
*Areca catechu* L.	Fruit	295.79 ± 11.97	2.62 ± 0.36
*Carissa carandas* L.	Fruit	14.06 ± 1.55	2.83 ± 0.78
*Centella asiatica* (L.) Urb.	Leaf	15.26 ± 0.76	11.25 ± 2.87
*Clitoria macrophylla* Wall.	Flower	64.85 ± 2.81	14.74 ± 2.71
*Clitoria ternatea* L.	Flower	25.60 ± 2.19	10.33 ± 2.31
*Eleutherine americana* (Aubl.) Merr.	Rhizome	73.73 ± 1.87	2.61 ± 0.42
*Glochidion zeylanicum* (Gaertn.) A. Juss.	Leaf	320.14 ± 7.95	52.54 ± 7.25
*Hylocereus undatus* (Haw.) Britt. Rose.	Peel	70.39 ± 4.57	22.20 ± 3.20
*Mangifera caloneura* Kurz.	Leaf	210.99 ± 10.40	84.48 ± 18.32
*Piper nigrum* L.	Seed	47.86 ± 2.27	3.46 ± 0.70
*Pithecellobium dulce* (Roxb.) Benth.	Peel	61.82 ± 0.61	28.01 ± 4.39
*Senna alata* (L.) Roxb.	Leaf	45.36 ± 1.15	10.24 ± 2.52
*Streblus asper* Lour.	Bark	18.02 ± 0.30	2.72 ± 0.89
*Streblus asper* Lour.	Leaf	43.58 ± 2.40	3.30 ± 1.50
*Zingiber cassumunar* Roxb.	Rhizome	44.63 ± 0.61	4.85 ± 0.98
*Zingiber officinale* Roscoe.	Rhizome	139.94 ± 2.27	2.86 ± 0.74

Values show mean ± standard deviation (SD) of at least three independent experiments; GAE = gallic acid equivalent; QE = quercetin equivalent.

**Table 3:** DPPH radical scavenging activity of Thai plants.

Scientific Name	Part Used	DPPH Radical Scavenging		
		Scavenging Activity (%)	mg VCEAC/g Dry Weight Extract	IC_50_ (µg/mL)
*Anacardium occidentale* L.	Leaf	89.01 ± 1.51	387.43 ± 13.97	18.68 ± 0.59
*Areca catechu* L.	Fruit	88.12 ± 5.04	627.64 ± 8.94	9.85 ± 0.91
*Carissa carandas* L.	Fruit	12.76 ± 1.13	16.63 ± 2.32	>100
*Centella asiatica* (L.) Urb.	Leaf	15.38 ± 0.93	19.52 ± 1.48	>100
*Clitoria macrophylla* Wall.	Flower	9.01 ± 1.43	10.03 ± 1.50	>100
*Clitoria ternatea* L.	Flower	6.40 ± 0.45	7.49 ± 0.29	>100
*Eleutherine americana* (Aubl.) Merr.	Rhizome	45.58 ± 7.14	53.49 ± 6.97	188.05 ± 43.01
*Glochidion zeylanicum* (Gaertn.) A. Juss.	Leaf	93.18 ± 0.64	1154.54 ± 36.19	6.56 ± 0.46
*Hylocereus undatus* (Haw.) Britt. Rose.	Peel	26.57 ± 2.03	33.91 ± 2.07	>100
*Mangifera caloneura* Kurz.	Leaf	90.61 ± 3.27	289.44 ± 10.68	20.89 ± 2.27
*Piper nigrum* L.	Seed	14.71 ± 1.72	18.74 ± 2.78	>100
*Pithecellobium dulce* (Roxb.) Benth.	Peel	46.84 ± 3.68	55.09 ± 2.72	120.84 ± 25.33
*Senna alata* (L.) Roxb.	Leaf	27.16 ± 4.56	33.12 ± 4.28	>100
*Streblus asper* Lour.	Bark	11.93 ± 2.26	13.14 ± 1.76	>100
*Streblus asper* Lour.	Leaf	28.53 ± 2.63	30.94 ± 2.05	>100
*Zingiber cassumunar* Roxb.	Rhizome	33.37 ± 4.70	40.61 ± 4.99	253.63 ± 24.05
*Zingiber officinale* Roscoe.	Rhizome	71.73 ± 5.29	82.22 ± 6.68	67.21 ± 13.31

Values show mean ± standard deviation (SD) of at least three independent experiments; IC_50_ is the concentration at which the 50% scavenging activity is observed; VCEAC = Vitamin C equivalent antioxidant capacity.

**Table 4:** ABTS radical scavenging activity of Thai plants.

Scientific Name	Part Used	ABTS Radical Scavenging		
		Scavenging Activity (%)	mg VCEAC/g Dry Weight Extract	IC_50_ (µg/mL)
*Anacardium occidentale* L.	Leaf	99.23 ± 0.29	675.44 ± 65.66	8.64 ± 0.66
*Areca catechu* L.	Fruit	99.31 ± 0.32	837.47 ± 44.16	5.14 ± 1.42
*Carissa carandas* L.	Fruit	13.93 ± 1.03	12.41 ± 1.97	>100
*Centella asiatica* (L.) Urb.	Leaf	18.86 ± 4.81	16.98 ± 4.73	324.22 ± 46.00
*Clitoria macrophylla* Wall.	Flower	38.27 ± 7.24	34.78 ± 5.01	>100
*Clitoria ternatea* L.	Flower	26.48 ± 2.24	26.35 ± 3.28	>100
*Eleutherine americana* (Aubl.) Merr.	Rhizome	95.62 ± 6.13	213.63 ± 9.12	20.23 ± 4.72
*Glochidion zeylanicum* (Gaertn.) A. Juss.	Leaf	99.37 ± 0.21	1184.59 ± 51.41	3.76 ± 0.79
*Hylocereus undatus* (Haw.) Britt. Rose.	Peel	82.16 ± 2.17	81.73 ± 4.78	44.23 ± 5.13
*Mangifera caloneura* Kurz.	Leaf	98.88 ± 1.55	531.29 ± 26.11	9.31 ± 0.85
*Piper nigrum* L.	Seed	33.28 ± 4.72	30.93 ± 4.13	150.35 ± 34.82
*Pithecellobium dulce* (Roxb.) Benth.	Peel	83.99 ± 6.01	79.65 ± 4.36	49.52 ± 7.01
*Senna alata* (L.) Roxb.	Leaf	64.95 ± 7.32	61.92 ± 5.38	52.20 ± 2.94
*Streblus asper* Lour.	Bark	23.55 ± 2.92	23.29 ± 1.33	>100
*Streblus asper* Lour.	Leaf	65.60 ± 6.47	59.33 ± 6.67	66.36 ± 7.89
*Zingiber cassumunar* Roxb.	Rhizome	73.36 ± 5.89	69.88 ± 5.88	43.60 ± 5.49
*Zingiber officinale* Roscoe.	Rhizome	94.07 ± 3.44	175.47 ± 16.28	22.76 ± 9.79

Values show mean ± standard deviation (SD) of at least three independent experiments; IC_50_ is the concentration at which the 50% scavenging activity is observed; VCEAC = Vitamin C equivalent antioxidant capacity.

**Table 5:** Elastase inhibitory activity of Thai plants.

Scientific Name	Part Used	Elastase Inhibition (%)		
		0.5 mg/mL	0.1 mg/mL	IC_50_ (µg/mL)
*Anacardium occidentale* L.	Leaf	84.78 ± 2.16	-	18.21 ± 4.91
*Areca catechu* L.	Fruit	88.31 ± 0.41	-	117.07 ± 21.71
*Carissa carandas* L.	Fruit	nd	-	-
*Centella asiatica* (L.) Urb.	Leaf	nd	-	-
*Clitoria macrophylla* Wall.	Flower	9.85 ± 2.26	-	>500
*Clitoria ternatea* L.	Flower	nd	-	-
*Eleutherine americana* (Aubl.) Merr.	Rhizome	nd	-	-
*Glochidion zeylanicum* (Gaertn.) A. Juss.	Leaf	87.43 ± 3.80	-	47.94 ± 24.75
*Hylocereus undatus* (Haw.) Britt. Rose.	Peel	nd	-	-
*Mangifera caloneura* Kurz.	Leaf	na	13.75 ± 1.61	>100
*Piper nigrum* L.	Seed	na	9.05 ± 0.09	>100
*Pithecellobium dulce* (Roxb.) Benth.	Peel	22.87 ± 2.92	-	>500
*Senna alata* (L.) Roxb.	Leaf	73.95 ± 1.46	-	82.25 ± 19.99
*Streblus asper* Lour.	Bark	1.33 ± 0.89	-	> 500
*Streblus asper* Lour.	Leaf	na	35.74 ± 0.94	153.28 ± 2.39
*Zingiber cassumunar* Roxb.	Rhizome	na	3.46 ± 1.29	>100
*Zingiber officinale* Roscoe.	Rhizome	na	nd	-
EGCG (0.1 mg/mL)		-	45.27 ± 3.36	-

Values show mean ± standard deviation (SD) of at least three independent experiments; IC_50_ is the concentration at which the 50% inhibition level is observed; EGCG = epigallocatechin gallate; nd = not detectable; na = not applicable (high background); - = not tested.

**Table 6:** Tyrosinase inhibitory activity of Thai plants.

Scientific Name	Part Used	Tyrosinase Inhibition (%)		
		1 mg/mL	0.1 mg/mL	IC_50_ (µg/mL)
*Anacardium occidentale* L.	Leaf	81.01 ± 2.96	-	307.66 ± 65.12
*Areca catechu* L.	Fruit	75.38 ± 1.57	-	85.73 ± 8.26
*Carissa carandas* L.	Fruit	15.00 ± 1.21	-	>1000
*Centella asiatica* (L.) Urb.	Leaf	13.57 ± 1.23	-	>1000
*Clitoria macrophylla* Wall.	Flower	27.36 ± 7.95	-	>1000
*Clitoria ternatea* L.	Flower	10.02 ± 1.61	-	>1000
*Eleutherine americana* (Aubl.) Merr.	Rhizome	45.10 ± 1.59	-	>1000
*Glochidion zeylanicum* (Gaertn.) A. Juss.	Leaf	91.51 ± 5.39	-	76.00 ± 4.31
*Hylocereus undatus* (Haw.) Britt. Rose.	Peel	15.79 ± 0.84	-	>1000
*Mangifera caloneura* Kurz.	Leaf	76.12 ± 3.98	-	457.63 ± 71.73
*Piper nigrum* L.	Seed	4.80 ± 1.31	-	>1000
*Pithecellobium dulce* (Roxb.) Benth.	Peel	nd	-	-
*Senna alata* (L.) Roxb.	Leaf	12.94 ± 2.73	-	>1000
*Streblus asper* Lour.	Bark	9.85 ± 1.14	-	>1000
*Streblus asper* Lour.	Leaf	5.11 ± 3.88	-	>1000
*Zingiber cassumunar* Roxb.	Rhizome	na	3.98 ± 0.54	>100
*Zingiber officinale* Roscoe.	Rhizome	na	21.28 ± 2.53	>100
KA (0.02 mg/mL)		-	68.35 ± 1.22	-

Values show mean ± standard deviation (SD) of at least three independent experiments; IC_50_ is the concentration at which the 50% inhibition level is observed; KA = kojic acid; nd = not detectable; na = not applicable (high background); - = not tested.

## Data Availability

Not applicable.

## Supplementary Materials

The following supporting information can be downloaded at: https://www.mdpi.com/article/10.3390/plants12010065/s1, Figure S1: correlation analysis between phytochemical contents and antioxidant capacities among the studied plants; Table S1: the percent inhibition of elastase activity by Thai plants at various concentrations; Table S2: the percent inhibition of tyrosinase activity by Thai plants at various concentrations; Table S3: list of compounds derived from three most effective plants on inhibition against elastase and/or tyrosinase; Table S4: molecular docking results between phytochemical compounds and the binding site of elastase (3HGP); Table S5: molecular docking results between phytochemical compounds and the binding site of tyrosinase (2Y9X). References [25,26,27,28,30,31,32,42,50,51,52,53,54,55,56,57,58,59,60,61] are cited in the Supplementary Materials.
