# Supplementary material for: Antioxidant and Anti-Skin Aging Potential of Selected Thai Plants: In Vitro Evaluation and In Silico Target Prediction
Source: Plants (Basel). 2022 Dec 22;12(1):65. doi: 10.3390/plants12010065 (PMC9823845; doi:10.3390/plants12010065)
Supplement: Supplementary file 1 [file plants-12-00065-s001.zip › plants-2035503-supplementary.pdf]

Supplementary Materials

Antioxidant and Anti-Skin Aging Potential of Selected Thai Plants: In Vitro Evaluation and In Silico Target Prediction

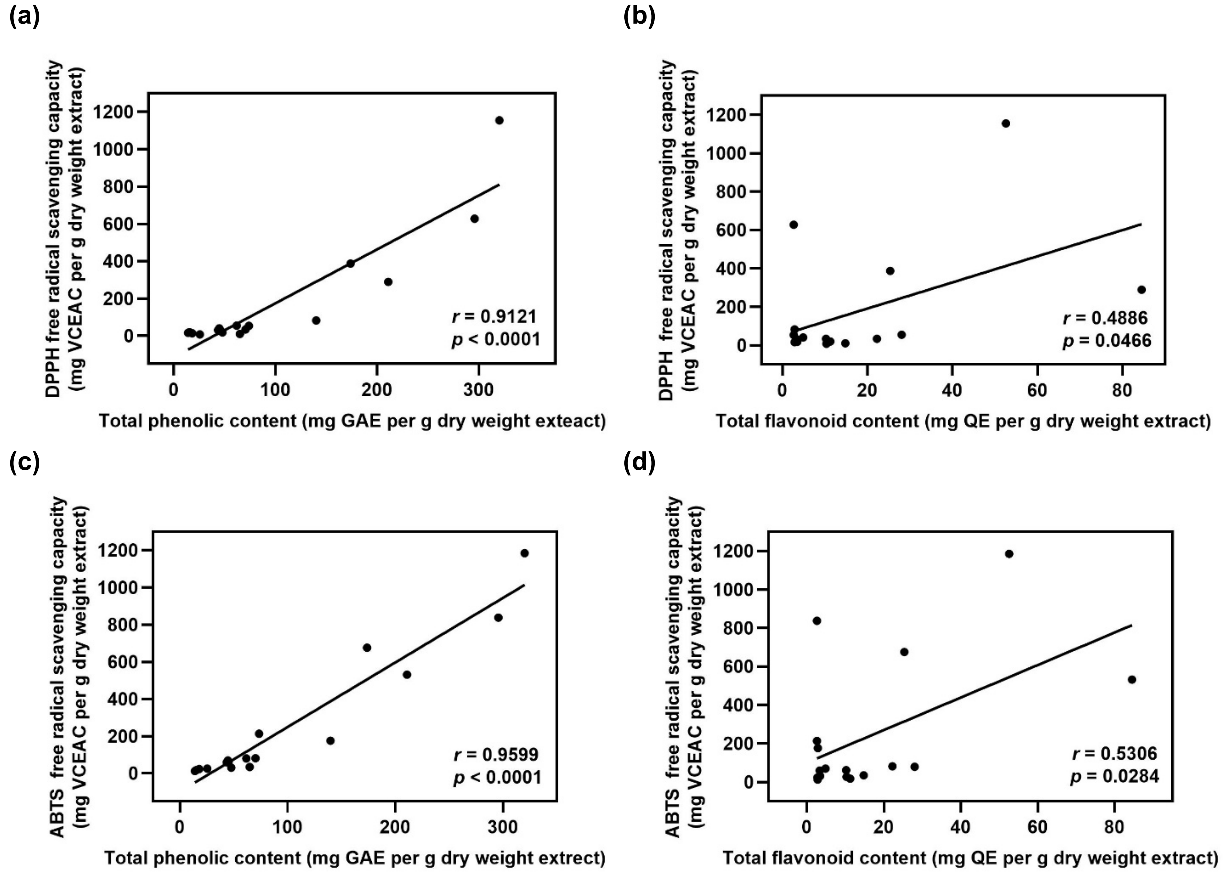

Figure S1. Correlation analysis between phytochemical contents and antioxidant capacities among the studied plants. The correlations are expressed as Pearson's correlation coefficients (r) between **(a)** total phenolic content and DPPH free radical scavenging activity, **(b)** total phenolic content and ABTS free radical scavenging activity, **(c)** total flavonoid content and DPPH free radical scavenging activity, **(d)** total phenolic content and ABTS free radical scavenging activity.

Table S1. The percent inhibition of elastase activity by Thai plants at various concentrations.

Anacardium occidentale L. (Leaf)	
**Concentration (µg/ml)**	**Inhibition (%)**
500.00	84.78 ± 2.16
100.00	78.09 ± 3.91
50.00	71.73 ± 6.58
25.00	62.23 ± 7.48
12.50	36.87 ± 10.05
6.25	7.90 ± 4.67
***Areca catechu* L. (Fruit)**	
**Concentration (µg/ml)**	**Inhibition (%)**
500.00	88.31 ± 0.41
250.00	78.08 ± 4.51
125.00	52.35 ± 10.44
62.50	28.66 ± 2.80
31.25	13.75 ± 1.30
15.63	8.49 ± 2.19
7.81	8.57 ± 4.85
***Glochidion zeylanicum* (Gaertn.) A. Juss. (Leaf)**	
**Concentration (µg/ml)**	**Inhibition (%)**
500.00	87.43 ± 3.80
100.00	66.00 ± 10.67
50.00	54.16 ± 10.29
25.00	34.94 ± 18.68
12.50	25.03 ± 16.41
6.25	20.66 ± 9.82
***Senna alata* (L.) Roxb. (Leaf)**	
**Concentration (µg/ml)**	**Inhibition (%)**
500.00	73.95 ± 1.46
250.00	65.61 ± 1.35
125.00	57.85 ± 1.81
62.50	44.81 ± 6.25
31.25	29.04 ± 10.78
15.63	19.75 ± 12.27
7.81	16.19 ± 12.76
***Streblus asper* Lour. (Leaf)**	
**Concentration (µg/ml)**	**Inhibition (%)**
400.00	75.25 ± 8.25
200.00	58.30 ± 2.26
100.00	35.74 ± 0.94
50.00	13.01 ± 4.66

Values show mean ± standard deviation (SD) of at least three independent experiments.

Table S2. The percent inhibition of tyrosinase activity by Thai plants at various concentrations.

Anacardium occidentale L. (Leaf)	
**Concentration (µg/ml)**	**Inhibition (%)**
2000.00	84.41 ± 4.30
1000.00	81.01 ± 2.96
500.00	66.13 ± 8.01
250.00	41.10 ± 11.78
125.00	18.12 ± 10.02
62.50	8.27 ± 2.64
31.25	2.90 ± 0.79
***Areca catechu* L. (Fruit)**	
**Concentration (µg/ml)**	**Inhibition (%)**
1000.00	75.38 ± 1.57
500.00	72.94 ± 1.48
250.00	66.88 ± 3.60
125.00	60.94 ± 3.61
62.50	41.82 ± 1.43
31.25	30.14 ± 9.51
15.63	24.86 ± 7.30
***Glochidion zeylanicum* (Gaertn.) A. Juss. (Leaf)**	
**Concentration (µg/ml)**	**Inhibition (%)**
500.00	90.43 ± 1.57
250.00	87.70 ± 1.19
125.00	74.47 ± 1.47
62.50	38.05 ± 4.30
31.25	5.45 ± 4.95
***Mangifera caloneura* Kurz. (Leaf)**	
**Concentration (µg/ml)**	**Inhibition (%)**
1000.00	76.12 ± 3.98
500.00	52.96 ± 5.12
250.00	34.55 ± 4.40
125.00	21.92 ± 1.46
62.50	12.53 ± 3.71

Values show mean ± standard deviation (SD) of at least three independent experiments.

Table S3. List of compounds derived from three most effective plants on inhibition against elastase and/or tyrosinase.

Compound	Structure	Source	Reference
Agathisflavone	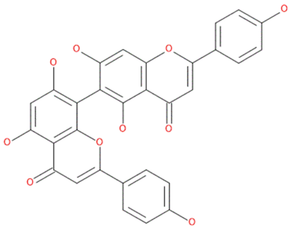	*Anacardium occidentale*	[26]
Aloe-emodin	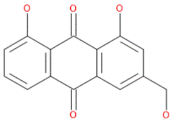	*Senna alata*	[50,57]
Amentoflavone	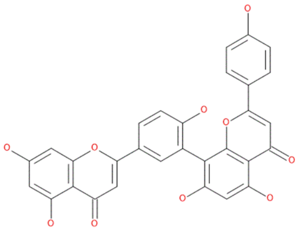	*Anacardium occidentale*	[25]
Apigenin	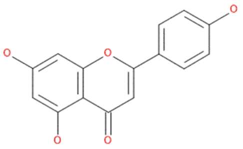	*Senna alata*	[55]
Arecaidine	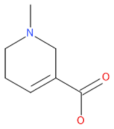	*Areca catechu*	[30,51]
Arecatannin A1	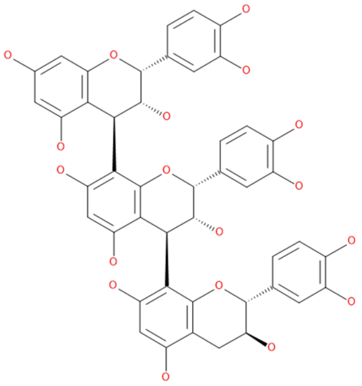	*Areca catechu*	[31]

Arecatannin B1	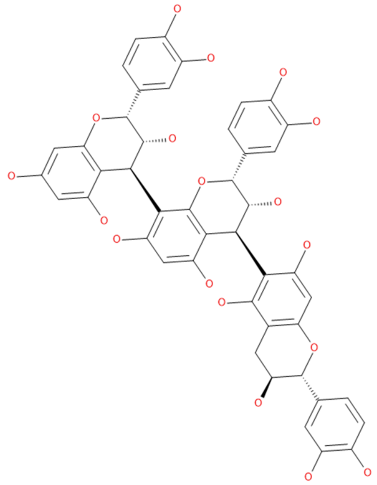	*Areca catechu*	[31]
Arecoline	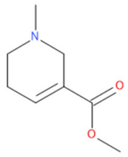	*Areca catechu*	[30,51]
Benzoic acid	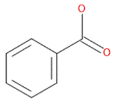	*Glochidion zeylanicum*	[42]
Caffeic acid	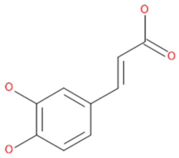	*Areca catechu*	[30]
Catechin	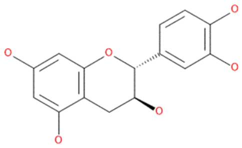	*Areca catechu*, *Anacardium occidentale*, *Glochidion zeylanicum*	[25,27,30-32,42,53,59]
Chrysoeriol	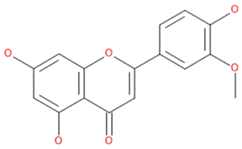	*Senna alata*, *Areca catechu*	[52,61]
Coumarin	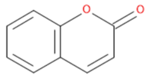	*Areca catechu*	[30]
Diosmetin	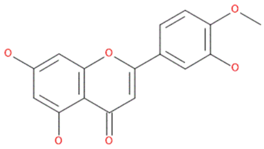	*Senna alata*	[57]

Emodin	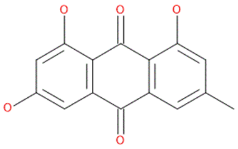	*Senna alata*	[50,54,57]
Epicatechin	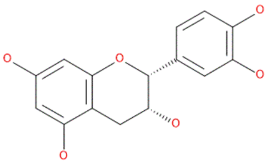	*Areca catechu*, *Anacardium occidentale*	[25,30,31, 53,59,60]
Epigallocatechin	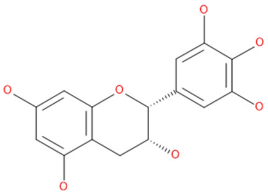	*Areca catechu*	[30,53]
Epigallocatechin gallate	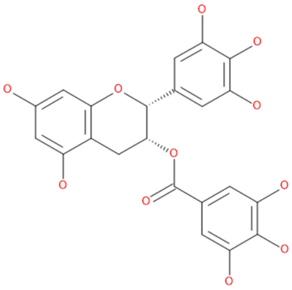	*Areca catechu*	[53]
Ferulic acid	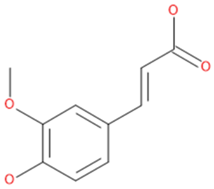	*Areca catechu*	[30]
Gallic acid	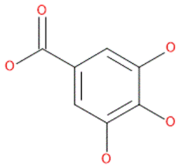	*Areca catechu*, *Anacardium occidentale*, *Glochidion zeylanicum*	[27,30,32, 42,53]
Gallocatechin	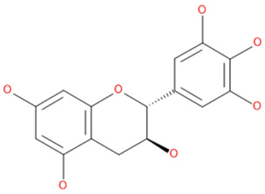	*Areca catechu*	[30]

Gallocatechin gallate	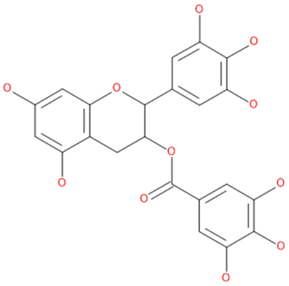	*Areca catechu*	[30]
Ginkgolide B	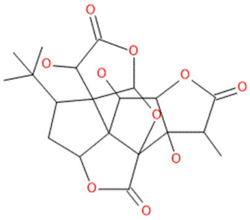	*Glochidion zeylanicum*	[42]
Glycitin	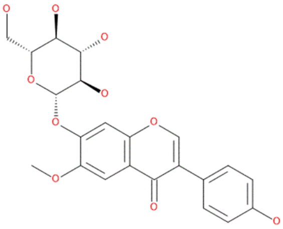	*Glochidion zeylanicum*	[42]
Guvacine	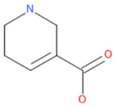	*Areca catechu*	[30,51]
Guvacoline	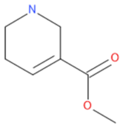	*Areca catechu*	[30,51]
Hexanedioic acid	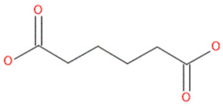	*Glochidion zeylanicum*	[42]
Isoquercetin	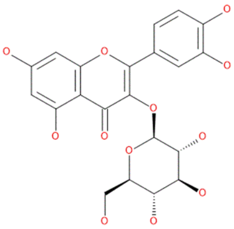	*Anacardium occidentale*	[27]
Isorhamnetin	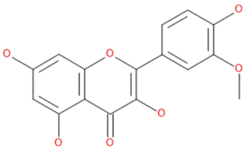	*Areca catechu*	[61]

Isorhamnetin-3-O-galactoside	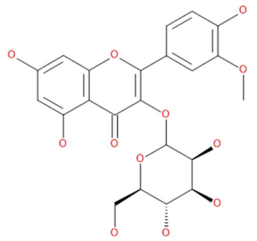	*Areca catechu*	[30]
Jacareubin	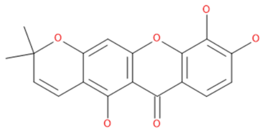	*Areca catechu*	[61]
Kaempferol	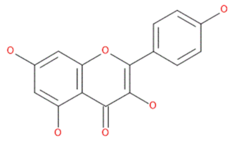	*Areca catechu*, *Senna alata*	[30,50,52, 57,58]
Kaempferol 3-O-beta-L-glucopyranoside	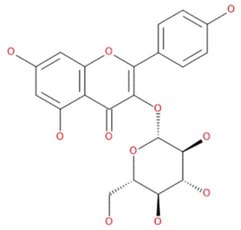	*Senna alata*	[52]
Kaempferol 3-O-gentiobioside	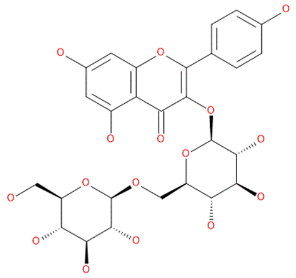	*Senna alata*	[28]
Kaempferol-3-O-glucoside	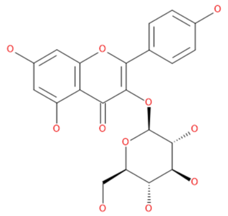	*Areca catechu*	[30]
Linoleic acid	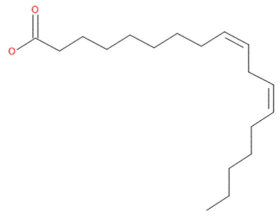	*Senna alata*	[54]

L-proline	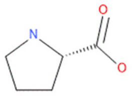	*Glochidion zeylanicum*	[42]
Luteolin	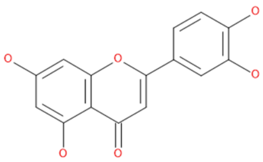	*Areca catechu*	[61]
Myricetin	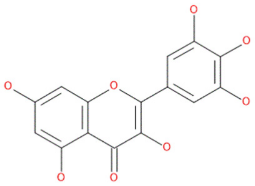	*Anacardium occidentale*	[25]
Myristic acid	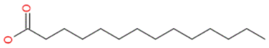	*Senna alata*	[54]
Naringenin	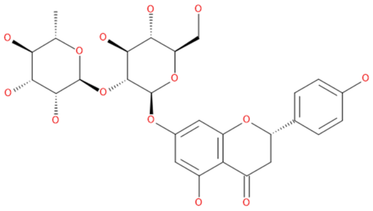	*Areca catechu*	[30]
Naringin	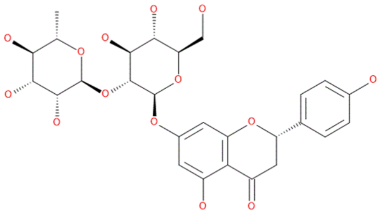	*Senna alata*	[55]
n-dotriacontanol	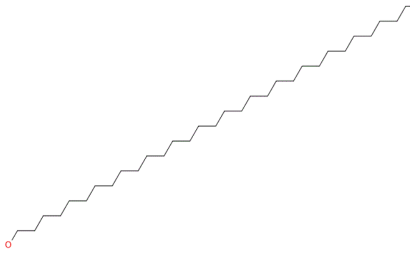	*Senna alata*	[52]
n-triacontanol	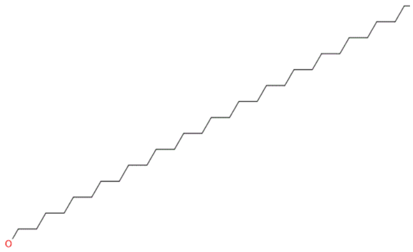	*Senna alata*	[52]

o-Coumaric acid	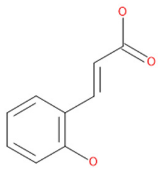	*Areca catechu*	[30]
Octadecatrienoic acid	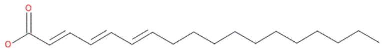	*Glochidion zeylanicum*	[42]
Oleic acid	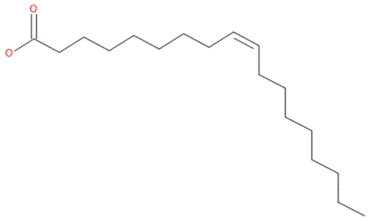	*Senna alata*	[54]
Oxyresveratrol	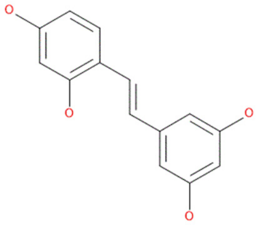	*Glochidion zeylanicum*	[42]
Palmitic acid	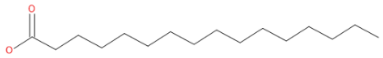	*Anacardium occidentale*, *Glochidion zeylanicum,* *Senna alata*	[32,42,52, 54]
Pentadecanoic acid	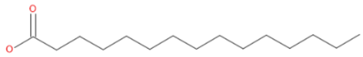	*Glochidion zeylanicum*	[42]
Phytol	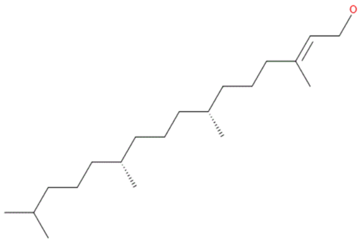	*Glochidion zeylanicum*	[42]
Quercetin	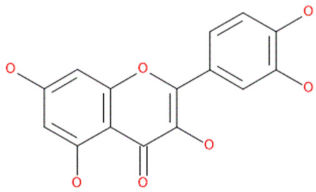	*Areca catechu*, *Anacardium occidentale*, *Glochidion zeylanicum,* *Senna alata*	[25,26,30, 32,42,52]

Quercetin-3-O-galactoside	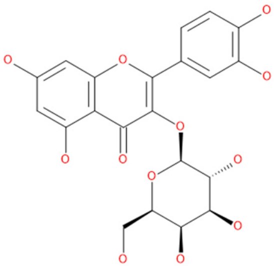	*Areca catechu*	[30]
Quinic acid	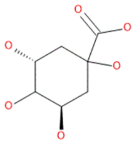	*Glochidion zeylanicum*	[42]
Resveratrol 4′-methyl ether	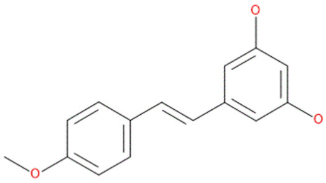	*Glochidion zeylanicum*	[42]
Rhein	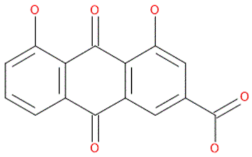	*Senna alata*	[50,54,56, 58]
Rutin	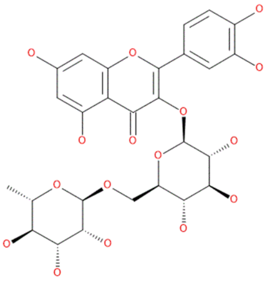	*Anacardium occidentale*	[26,27]
Stearic acid	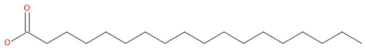	*Senna alata*	[52,54]
Syringic acid	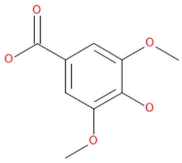	*Areca catechu*	[60]
Tetramer of proanthocyanidin	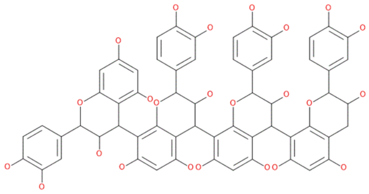	*Anacardium occidentale*	[25]

Vanillin	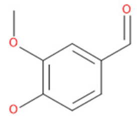	*Areca catechu*	[30]
α-linolenic acid	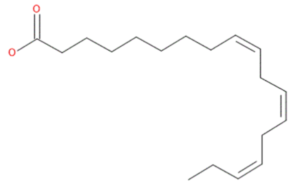	*Anacardium occidentale*, *Glochidion zeylanicum*	[32,42]

Table S4. Molecular docking results between phytochemical compounds and the binding site of elastase (3HGP).

No.	Compound	MW	Binding energy (kcal/mol)	Inhibition Constant	Number of hydrogen bond	Amino acid interaction	Bond length (Å)
	FRW (original inhibitor)	513.51	-7.14	5.80 µM	9	A:HIS57	2.52350
						A:HIS57	2.75545
						A:THR175	2.24654
						A:GLY193	2.33614
						A:GLY193	2.47173
						A:SER195	2.46901
						A:VAL216	2.18525
						A:VAL216	2.21136
						A:GLN192	2.64759
	Epigallocatechin gallate (positive control)	458.37	-9.69	79.13 nM	16	A:THR41	2.57071
						A:ARG61	2.53699
						A:ARG61	2.34579
						A:CYS191	2.70525
						A:GLY193	2.13234
						A:GLY193	1.99259
						A:ASP194	2.85786
						A:ASP194	2.95908
						A:SER195	2.27809
						A:SER195	2.85192
						A:VAL216	2.32268
						A:SER217	2.68922
						A:CYS191	3.08133
						A:CYS191	2.48834
						A:THR41	3.24692
						A:THR41	3.04781
1	Tetramer of proanthocyanidin	1155.02	-11.95	1.75 nM	18	A:HIS57	2.78926
						A:VAL99	2.06369
						A:GLY193	2.67412
						A:SER195	2.67016
						A:VAL216	2.58477
						A:ARG217A	2.14988
						A:ASP97	2.49907
						A:VAL216	3.23714
						A:CYS191	3.28470
						A:SER195	2.59907
						A:SER195	2.58138

						A:THR41	2.90533
						A:CYS42	3.17172
						A:CYS42	2.90763
						A:HIS57	3.36115
						A:CYS58	3.15629
						A:ASP97	2.52302
						A:CYS58	2.85453
2	Amentoflavone	538.46	-11.81	2.21 nM	7	A:VAL99	2.04173
						A:GLY193	1.97784
						A:ASP194	2.81399
						A:SER195	2.08605
						A:VAL216	2.95069
						A:VAL216	3.13572
						A:ARG217A	3.56022
3	Rutin	610.52	-10.12	38.08 nM	12	A:VAL99	2.15421
						A:GLY193	2.01978
						A:ASP194	2.68737
						A:SER195	2.03458
						A:ARG217A	2.89183
						A:ARG217A	2.16727
						A:ARG217A	3.00142
						A:ARG217A	2.77342
						A:ASP97	3.13763
						A:CYS191	2.70211
						A:GLN192	2.85245
						A:ASP97	2.94995
4	Agathisflavone	538.46	-9.92	53.93 nM	0		
5	Kaempferol 3-O-gentiobioside	610.52	-9.73	73.92 nM	8	A:HIS57	3.05099
						A:VAL99	2.83989
						A:ARG217A	1.83288
						A:ARG217A	2.67981
						A:ASP97	2.69662
						A:ASP98	3.35512
						A:ASP98	3.27145
						A:PHE215	4.19666
6	Kaempferol 3-O-beta-L-glucopyranoside	448.38	-9.36	136.73 nM	9	A:VAL99	2.35829
						A:GLY193	2.07372
						A:ASP194	2.84954
						A:SER195	2.16655
						A;VAL216	2.54727
						A:ASP97	3.15783

						A:ASP97	3.22664
						A:PHE215	2.94950
						A:VAL216	3.24286
7	Isoquercetin	464.38	-9.26	162.78 nM	9	A:ARG61	2.97515
						A:ARG61	2.54804
						A:ARG61	2.90630
						A:GLY193	2.03511
						A:ASP194	2.58534
						A:SER195	2.03300
						A:VAL216	2.68435
						A:VAL216	2.75760
						A:PHE215	2.95244
8	Naringin	580.53	-9.16	192.85 nM	16	A:ARG61	2.66162
						A:ARG61	3.06606
						A:GLN192	2.03957
						A:GLY193	1.99418
						A:GLY193	2.19799
						A:ASP194	2.67659
						A:SER195	3.05382
						A:SER195	2.27226
						A:CYS191	2.79285
						A:VAL216	2.79621
						A:SER214	2.75276
						A:ARG61	3.35884
						A:ARG217A	3.35938
						A:SER217	3.02352
						A:HIS57	2.98501
						A:HIS57	3.97829
9	Glycitin	446.40	-8.67	439.04 nM	10	A:HIS57	2.85020
						A:ALA99A	1.87321
						A:CYS191	2.94348
						A:ASP194	2.72853
						A:SER195	2.47003
						A:CYS191	2.54852
						A:ASP98	3.36807
						A:ASP97	3.09022
						A:VAL216	2.84750
						A:VAL216	3.13688
10	Myricetin	318.24	-8.53	561.70 nM	8	A:GLY193	2.07707
						A:ASP194	2.86297
						A:SER195	2.21726

						A:VAL216	2.50270
						A:ARG217A	3.06755
						A:SER217	3.21367
						A:ARG217A	3.73332
						A:VAL216	3.19504
11	Epicatechin	290.27	-8.47	618.86 nM	8	A:HIS57	3.01160
						A:GLY193	2.04469
						A:ASP194	2.83623
						A:SER195	2.17262
						A:VAL216	2.60128
						A:SER214	2.84466
						A:VAL216	3.24085
						A:HIS57	4.04784
12	Quercetin	302.24	-8.39	707.97 nM	6	A:GLY193	2.07626
						A:ASP194	2.85099
						A:SER195	2.20819
						A:VAL216	2.49512
						A:SER217	2.95390
						A:VAL216	3.20176
13	Catechin	290.27	-8.31	812.28 nM	5	A:GLY193	2.04521
						A:ASP194	2.83797
						A:SER195	2.17203
						A:VAL216	2.62754
						A:VAL216	3.09925
14	Ginkgolide B	424.40	-8.28	855.21 nM	7	A:HIS57	2.16026
						A:GLN192	2.28679
						A:GLY193	2.44387
						A:SER195	2.42828
						A:VAL216	2.77862
						A:VAL216	2.26792
						A:GLN192	3.22896
15	Chrysoeriol	300.26	-8.15	1.06 µM	5	A:GLY193	2.09633
						A:ASP194	2.86127
						A:SER195	2.24263
						A:VAL216	2.43954
						A:VAL216	3.25443
16	Diosmetin	300.26	-8.14	1.08 µM	6	A:GLY193	2.09905
						A:ASP194	2.87765
						A:SER195	2.25027
						A:VAL216	2.43233
						A:SER217	3.02011

						A:VAL216	3.28497
17	Kaempferol	286.24	-8.14	1.09 µM	5	A:GLY193	2.08399
						A:ASP194	2.85703
						A:SER195	2.22547
						A:VAL216	2.48090
						A:VAL216	3.15619
18	Apigenin	270.24	-7.98	1.42 µM	5	A:GLY193	2.08842
						A:ASP194	2.85822
						A:SER195	2.23099
						A:VAL216	2.45696
						A:VAL216	3.28784
19	Oxyresveratrol	244.24	-7.75	2.08 µM	5	A:GLY193	1.98392
						A:ASP194	2.50978
						A:SER195	1.99662
						A:VAL216	2.00620
						A:VAL216	2.70284
20	Emodin	270.24	-7.67	2.39 µM	5	A:GLY193	2.83596
						A:GLY193	1.98415
						A:ASP194	2.49870
						A:SER195	1.99120
						A:VAL216	2.06841
21	Aloe-emodin	270.24	-7.65	2.47 µM	7	A:GLY193	2.80483
						A:GLY193	2.00453
						A:ASP194	2.49731
						A:SER195	2.01419
						A:VAL216	2.13841
						A:PHE215	2.98478
						A:PHE215	3.14578
22	Rhein	284.22	-7.35	4.11 µM	10	A:ARG61	2.09898
						A:ARG61	2.07925
						A:GLY193	1.86295
						A:GLY193	2.02935
						A:ASP194	2.95764
						A:SER195	3.08050
						A:SER195	2.23777
						A:CYS191	2.88867
						A:THR41	2.61189
						A:CYS42	3.68229
23	Resveratrol 4′-methyl ether	242.27	-7.12	6.05 µM	4	A:SER195	2.03502
						A:GLY193	2.00145
						A:ASP194	2.56312

						A:ARG217A	2.54819
24	Gallic acid	170.12	-5.92	45.66 µM	6	A:GLY193	2.08325
						A:ASP194	2.63537
						A:SER195	2.63215
						A:SER195	2.15255
						A:VAL216	2.09269
						A:CYS191	2.89322
25	Octadecatrienoic acid	278.43	-5.70	65.93 µM	2	A:ARG217A	1.99352
						A:ARG217A	2.50481
26	Phytol	296.53	-5.53	88.41 µM	2	A:ARG217A	2.18541
						A:VAL216	3.16650
27	α-linolenic acid	278.43	-5.21	151.38 µM	2	A:ARG217A	2.41950
						A:ARG217A	2.12768
28	Quinic acid	192.17	-5.14	170.27 µM	7	A:GLY193	2.14684
						A:ASP194	2.27959
						A:SER195	2.09585
						A:VAL216	2.13330
						A:CYS191	2.47275
						A:CYS191	2.66879
						A:SER195	2.72310
29	Linoleic acid	280.45	-4.91	9.09 µM	1	A:ARG217A	2.45050
30	Oleic acid	282.46	-4.83	287.10 µM	1	A:ARG217A	2.09175
31	Palmitic acid	256.42	-4.58	438.97 µM	1	A:ARG217A	1.92107
32	Pentadecanoic acid	242.40	-4.45	543.69 µM	2	A:ARG217A	2.14678
						A:ARG217A	2.88731
33	Benzoic acid	122.12	-4.24	779.03 µM	1	A:GLY193	1.94803
34	Stearic acid	284.48	-4.24	782.65 µM	2	A:ARG217A	2.66437
						A:ARG217A	3.05706
35	Myristic acid	228.37	-4.22	802.18 µM	0		
36	L-proline	115.13	-3.87	1.46 mM	3	A:ARG61	2.71531
						A:ASP60	3.20048
						A:ASP60	3.49653
37	Hexanedioic acid	146.14	-3.74	1.80 mM	4	A:ARG217A	1.99619
						A:ARG217A	1.83030
						A:ARG217A	1.97210
						A:VAL216	2.99642
38	n-dotriacontanol	466.87	-3.18	4.67 mM	3	A:ARG61	3.19083
						A:HIS57	3.27872
						A:ASP97	3.67089
39	n-triacontanol	438.81	-2.26	21.96 mM	1	A:THR175	2.14124

Table S5. Molecular docking results between phytochemical compounds and the binding site of tyrosinase (2Y9X).

No.	Compound	MW	Binding energy (kcal/mol)	Inhibition Constant	Number of hydrogen bond	Amino acid interaction	Bond length (Å)
	Tropolone (original inhibitor)	122.12	-4.69	364.55 µM	1	A:HIS61	2.93039
	Kojic acid (positive control)	142.11	-4.59	430.35 µM	8	A:ASN81	2.08063
						A:HIS85	2.32738
						A:GLU322	2.13654
						A:GLU322	3.04204
						A:CYS83	2.90731
						A:ASN320	3.42295
						A:GLU322	3.50682
						A:HIS85	2.51019
1	o-Coumaric acid	164.16	-10.42	23.09 nM	4	A:HIS263	2.97779
						A:HIS296	3.00656
						A:MET280	2.94362
						A:HIS263	3.80402
2	Tetramer of proanthocyanidin	1155.02	-10.42	22.83 nM	8	A:ASN81	1.94291
						A:ASN81	2.77879
						A:HIS244	2.25277
						A:ARG268	2.89527
						A:GLU322	3.20562
						A:MET280	2.71019
						A:HIS263	3.58654
						A:HIS263	3.11783
3	Caffeic acid	180.16	-10.10	39.74 nM	4	A:HIS61	3.00165
						A:HIS263	3.35274
						A:HIS296	2.79428
						A:GLY281	3.27655
4	Ferulic acid	194.18	-10.00	46.75 nM	5	A:HIS61	3.04213
						A:HIS263	3.29735
						A:HIS296	2.80888
						A:GLY281	3.32939
						A:ASN260	3.13639
5	Arecatannin A1	866.77	-9.94	110.38 nM	10	A:ASN81	2.38646
						A:ARG268	3.05168
						A:GLY281	2.84476
						A:SER282	2.74566
						A:ALA246	2.90249

						A:ALA323	3.06850
						A:CYS83	2.57007
						A:PRO284	3.04284
						A:ASN81	3.22583
						A:HIS244	3.54321
6	Octadecatrienoic acid	278.43	-9.87	58.62 nM	2	A:HIS94	3.16054
						A:HIS296	2.82951
7	Gallic acid	170.12	-9.65	84.78 nM	5	A:SER282	3.06267
						A:HIS263	3.09352
						A:HIS296	2.81572
						A:MET280	3.15132
						A:ASN260	2.88933
8	Quinic acid	192.17	-9.63	88.03 nM	6	A:ASN260	3.18435
						A:MET280	2.74930
						A:HIS61	3.32578
						A:HIS263	3.55667
						A:HIS263	3.32810
						A:HIS85	2.93841
9	Syringic acid	198.17	-9.56	98.46 nM	4	A:HIS263	2.89886
						A:HIS296	3.13860
						A:MET280	3.35634
						A:GLU256	3.11772
10	Arecatannin B1	866.77	-9.52	104.57 nM	11	A:HIS85	2.74069
						A:HIS85	1.66258
						A:VAL248	2.01703
						A:ARG268	1.95436
						A:ALA246	2.65678
						A:ALA246	3.25979
						A:GLU322	3.39371
						A:MET257	3.34050
						A:CYS83	3.17367
						A:CYS83	2.92554
						A:HIS244	3.48197
11	Amentoflavone	538.46	-9.37	136.28 nM	6	A:ASN81	2.32368
						A:ASN81	2.09600
						A:GLU322	3.01671
						A:CYS83	2.80356
						A:HIS85	2.79983
						A:HIS263	3.30620
12	α-linolenic acid	278.43	-9.32	146.97 nM	2	A:HIS94	3.22968
						A:HIS94	3.83676

13	Benzoic acid	122.12	-9.26	163.00 nM	1	A:HIS94	3.36341
14	Pentadecanoic acid	242.40	-8.90	300.01 nM	2	A:HIS263	3.03186
						A:HIS296	2.88041
15	Palmitic acid	256.42	-8.86	318.21 nM	2	A:HIS61	2.79886
						A:HIS296	2.77369
16	Hexanedioic acid	146.14	-8.68	433.58 nM	3	A:HIS94	3.16234
						A:HIS94	3.76420
						A:HIS263	3.40059
17	Arecaidine	141.17	-8.59	502.24 nM	3	A:HIS263	2.92899
						A:HIS296	3.09977
						A:ASN260	3.00512
18	Gallocatechin gallate	458.37	-8.41	679.90 nM	9	A:ASN260	2.09290
						A:ARG268	2.17559
						A:VAL283	1.77894
						A:SER282	3.26428
						A:HIS61	3.51813
						A:HIS85	2.99297
						A:HIS259	2.97683
						A:ARG268	3.56983
						A:HIS263	3.39447
19	Agathisflavone	538.46	-8.23	934.62 nM	5	A:ASN81	2.58158
						A:ASN260	2.99061
						A:ASN260	3.75864
						A:HIS85	2.54527
						A:VAL283	2.89718
20	Epigallocatechin gallate	458.37	-8.08	1.19 µM	6	A:ASN260	2.28274
						A:SER282	3.03305
						A:MET280	3.02781
						A:HIS61	3.22370
						A:HIS259	3.11352
						A:HIS263	3.53154
21	Guvacine	127.14	-7.93	1.53 µM	0		
22	Isorhamnetin-3-O-galactoside	478.40	-7.78	1.97 µM	10	A:ARG268	2.61244
						A:ARG268	2.15888
						A:MET280	3.23812
						A:MET280	2.88616
						A:ASN260	2.74567
						A:HIS259	3.24854
						A:HIS263	3.50211
						A:PHE264	3.92479
						A:PHE264	3.89086

						A:HIS263	3.31541
23	Isoquercetin	464.38	-7.74	2.11 µM	7	A:VAL283	1.95856
						A:HIS259	3.58479
						A:ARG268	2.92003
						A:PRO284	3.18679
						A:GLY281	2.96963
						A:HIS263	3.04766
						A:HIS263	4.03417
24	Quercetin-3-O-galactoside	464.38	-7.67	2.37 µM	8	A:VAL283	1.85543
						A:SER282	3.32748
						A:HIS61	3.72991
						A:HIS259	3.40187
						A:ARG268	2.91088
						A:PRO284	3.11048
						A:GLY281	2.91699
						A:HIS263	3.14255
25	Rutin	610.52	-7.65	2.48 µM	7	A:HIS244	1.71048
						A:ASN260	2.93145
						A:GLY281	3.22566
						A:HIS61	3.65135
						A:HIS259	3.37472
						A:PRO284	3.47025
						A:HIS263	3.19461
26	Kaempferol-3-O-glucoside	448.38	-7.40	3.79 µM	7	A:ARG268	2.01129
						A:ARG268	2.11496
						A:ARG268	2.66378
						A:ARG268	1.79570
						A:VAL283	1.91848
						A:GLY281	2.78962
						A:ARG268	2.88437
27	Myricetin	318.24	-7.04	6.92 µM	8	A:HIS244	1.98915
						A:HIS244	2.87322
						A:HIS61	3.14519
						A:HIS85	3.25815
						A:HIS296	3.36954
						A:HIS61	3.08605
						A:HIS244	3.69317
						A:HIS263	3.77767
28	Gallocatechin	306.27	-6.94	8.21 µM	5	A:HIS244	1.84683
						A:HIS263	2.96751
						A:SER282	3.07269

						A:MET280	2.71186
						A:HIS244	3.94980
29	Catechin	290.27	-6.85	9.60 µM	3	A:ASN260	2.82593
						A:HIS61	2.90610
						A:PHE264	3.84711
30	L-proline	115.13	-6.85	9.59 µM	3	A:HIS263	2.82224
						A:HIS296	3.06908
						A:HIS259	3.74845
31	Quercetin	302.24	-6.78	10.76 µM	7	A:HIS244	2.13838
						A:HIS244	2.89602
						A:HIS61	3.16356
						A:HIS85	3.24592
						A:HIS296	3.36613
						A:HIS61	3.09307
						A:HIS244	3.80014
32	Epicatechin	290.27	-6.69	12.58 µM	5	A:ASN260	2.79798
						A:HIS263	2.887000
						A:VAL283	2.87757
						A:MET280	2.73467
						A:HIS85	2.90708
33	Epigallocatechin	306.27	-6.68	12.71 µM	5	A:HIS263	2.89927
						A:VAL283	2.86357
						A:MET280	2.74093
						A:HIS85	2.86647
						A:HIS263	3.62499
34	Jacareubin	326.30	-6.56	15.53 µM	3	A:GLY281	2.99526
						A:HIS85	2.95688
						A:HIS259	2.99294
35	Glycitin	446.40	-6.47	18.22 µM	4	A:VAL283	2.64301
						A:MET280	2.62857
						A:HIS61	2.89798
						A:HIS263	3.31631
36	Isorhamnetin	316.26	-6.47	18.03 µM	7	A:HIS244	2.54417
						A:HIS263	2.94940
						A:SER282	2.80771
						A:MET280	2.49056
						A:HIS85	2.85990
						A:HIS259	3.71081
						A:HIS263	3.83165
37	Luteolin	286.24	-6.44	18.97 µM	3	A:ASN260	2.99906
						A:MET280	3.36915

						A:HIS263	3.67221
38	Naringenin	272.25	-6.42	19.81 µM	4	A:SER282	2.50052
						A:MET280	2.57025
						A:HIS85	2.97900
						A:HIS259	3.20637
39	Kaempferol	286.24	-6.39	20.80 µM	6	A:HIS244	2.58376
						A:HIS263	2.93476
						A:SER282	2.81249
						A:MET280	2.48505
						A:HIS85	2.82651
						A:HIS259	3.58735
40	Chrysoeriol	300.26	-6.36	21.87 µM	3	A:ASN260	2.68258
						A:MET280	3.29794
						A:HIS263	3.58794
41	Ginkgolide B	424.40	-6.16	30.38 µM	3	A:HIS244	1.72866
						A:ARG268	3.09288
						A:ASN260	3.06936
42	Oxyresveratrol	244.24	-5.93	44.90 µM	3	A:ARG268	2.13044
						A:MET280	2.81813
						A:HIS263	3.77046
43	Coumarin	146.14	-5.70	66.40 µM	1	A:HIS259	3.30589
44	Resveratrol 4′-methyl ether	242.27	-5.66	71.36 µM	2	A:SER282	2.73416
						A:MET280	2.77858
45	Vanillin	152.15	-5.00	217.70 µM	2	A:ASN260	3.28389
						A:PHE264	3.77041
46	Guvacoline	141.17	-4.95	234.06 µM	6	A:ARG321	2.24586
						A:ARG321	1.70808
						A:HIS244	3.79383
						A:GLY86	3.15994
						A:GLU322	2.82850
						A:GLU322	3.10460
47	Phytol	296.53	-4.64	394.43 µM	0		
48	Arecoline	155.19	-4.56	450.69 µM	6	A:CYS83	3.57655
						A:HIS85	1.93297
						A:ALA323	3.23921
						A:THR84	3.69370
						A:ASN320	3.21035
						A:GLU322	3.59248
